# A Role of Myocardin Related Transcription Factor-A (MRTF-A) in Scleroderma Related Fibrosis

**DOI:** 10.1371/journal.pone.0126015

**Published:** 2015-05-08

**Authors:** Xu Shiwen, Richard Stratton, Joanna Nikitorowicz-Buniak, Bahja Ahmed-Abdi, Markella Ponticos, Christopher Denton, David Abraham, Ayuko Takahashi, Bela Suki, Matthew D. Layne, Robert Lafyatis, Barbara D. Smith

**Affiliations:** 1 Centre for Rheumatology and Connective Tissue Diseases, University College London, Royal Free Campus, London, United Kingdom; 2 Department of Biomedical Engineering, Boston University, Boston, Massachusetts, United States of America; 3 Department of Biochemistry, Boston University School of Medicine, Boston, Massachusetts, United States of America; 4 Rheumatology Department, Boston University School of Medicine, Boston, Massachusetts, United States of America; University of Alabama at Birmingham, UNITED STATES

## Abstract

In scleroderma (systemic sclerosis, SSc), persistent activation of myofibroblast leads to severe skin and organ fibrosis resistant to therapy. Increased mechanical stiffness in the involved fibrotic tissues is a hallmark clinical feature and a cause of disabling symptoms. Myocardin Related Transcription Factor-A (MRTF-A) is a transcriptional co-activator that is sequestered in the cytoplasm and translocates to the nucleus under mechanical stress or growth factor stimulation. Our objective was to determine if MRTF-A is activated in the disease microenvironment to produce more extracellular matrix in progressive SSc. Immunohistochemistry studies demonstrate that nuclear translocation of MRTF-A in scleroderma tissues occurs in keratinocytes, endothelial cells, infiltrating inflammatory cells, and dermal fibroblasts, consistent with enhanced signaling in multiple cell lineages exposed to the stiff extracellular matrix. Inhibition of MRTF-A nuclear translocation or knockdown of MRTF-A synthesis abolishes the SSc myofibroblast enhanced basal contractility and synthesis of type I collagen and inhibits the matricellular profibrotic protein, connective tissue growth factor (CCN2/CTGF). In MRTF-A null mice, basal skin and lung stiffness was abnormally reduced and associated with altered fibrillar collagen. MRTF-A has a role in SSc fibrosis acting as a central regulator linking mechanical cues to adverse remodeling of the extracellular matrix.

## Introduction

Scleroderma (systemic sclerosis, SSc), a severe connective tissue disease with a high mortality rate affecting 1 in 8,000 people, is characterized by progressive fibrosis of skin and internal organs [1]. The pathobiology of this disease includes vascular injury, autoimmunity, and inflammation culminating in fibrosis, which disrupts the architecture of the dermis and multiple internal organs through an accumulation of extracellular matrix (ECM) rich in type I collagen [1–4]. The clinical subset, diffuse cutaneous SSc, is most extensive with a more severe overall fibrotic phenotype than other clinical subtypes [5]. Although biomechanical measurements of skin stiffness vary depending on site, all sites in diffuse SSc patients are stiffer than healthy controls [6]. In forearm skin, Young’s modulus, a measure of stiffness, is 50–80 kPa in diffuse SSc compared to 4–12 kPa in healthy individuals [7]. Collagen fibrils are considered the primary ECM protein responsible for biomechanics of tissue [8]. Skin thickness and stiffness correlates with increased myofibroblasts [9] and large (90–120 nm) diameter collagen fibrils [10]. Skin thickening and tightness are the hallmark clinical changes of SSc, and severity of the skin fibrosis correlates with mortality and overall outcome.

Myofibroblasts that mediate fibrosis contain α-smooth muscle actin (SMA) and deposit ECM composed of collagen and matricellular proteins such as connective tissue growth factor (CCN2/CTGF) [1, 3, 11]. SMA is regulated at the transcriptional level by serum response factor (SRF) and co-activators of the myocardin family [12]. Myocardin, a potent nuclear transcriptional co-activator expressed in cardiac and smooth muscle lineages, is required for smooth muscle specific gene expression [13]. The myocardin-related transcription factors, MRTF-A (also called MKL1/MAL/BSAC) and MRTF-B (also called MKL2), are ubiquitously expressed [14]. Signals of stress, mechanical force and migration activate Rho GTPases resulting in actin cytoskeleton polymerization into stress fibers, permitting nuclear translocation of MRTFs which link actin dynamics with gene transcription [15–17]. Once in the nucleus, the myocardin family drives transcription of cytoskeleton genes including *SMA* as well as *CCN2* [16]. Myocardin family members interact with SRF as homo- or heterodimers to stimulate transcription via conserved CArG box DNA elements [18]. Our data demonstrates that MRTF-A, not other family members, drives collagen *(COL1A2)* transcription and this regulation is largely SRF independent [19]. MRTF-A nuclear translocation can be blocked by the pharmacological inhibitor, CCG-1423, [20, 21] originally described as a Rho inhibitor [22] thus blocking the Rho/SRF/MRTF pathway.

Transforming growth factor β (TGFβ)-induced myofibroblast differentiation is considered a key feature of SSc fibrosis [23]. Contractile myofibroblasts, as well as epithelial cells, have been shown to release TGFβ from latency associated peptide via an integrin dependent mechanism which leads to enhanced signaling via TGFβ receptor complexes [24, 25]. Exposure to TGFβ activates MRTF-A [26, 27] and MRTF-A has been described as a critical mediator of TGFβ-induced epithelial to mesenchymal transition (EMT) [28, 29] as well as endothelial to mesenchymal (EndMT) transition [30] resulting in myofibroblast like cells. MRTF-A has been implicated in mechanical sensing of ECM to determine cell fate decisions [15, 31–36]. In particular, matrix stiffness, tensional homeostasis, and mechanical force induce myofibroblast differentiation through MRTF-A [12, 37–40]. Previously, MRTF-A loss-of-function mice (KO) have been shown to be resistant to cardiac fibrosis [41], hypoxia induced pulmonary hypertension [42], bleomycin induced lung fibrosis [38], and skin fibrosis [43]. Fibroblasts from MRTF-A KO mice [44] produce less collagen which can be rescued by over-expressing MRTF-A [19]. In addition, the cells do not transcribe more collagen driven GFP in response to TGFβ [45]. These data suggest that following injury, exposure to activated TGFβ, or stress, MRTF-A is activated to induce myofibroblast differentiation and collagen production from multiple cell types. It has been proposed that there is a feed forward mechanism for progression of fibrosis [38] such that during fibrosis, cells continue to respond to matrix stiffening producing abnormal amounts of ECM.

Our hypothesis is that MRTF-A is progressively activated in SSc and that inhibition of MRTF-A nuclear translocation or expression may alter the progression of fibrosis in SSc and possibly reduce stiffness. Our data, represents the first examination of MRTF-A expression in human skin and demonstrates that MRTF-A expression in the nucleus is increased in multiple cell types within the SSc skin. Knockdown of MRTF-A and inhibition of MRTF-A nuclear translocation by CCG-1423 abrogates collagen and the fibrotic matricellular protein CCN2 synthesis and cell contraction by SSc fibroblasts. In addition, we show that the KO MRTF-A mice display decreased stiffness and mechanical properties in skin and lungs, through altered collagen fibrils.

## Materials and Methods

### Study subjects and ethical statements

**Fig 1:**
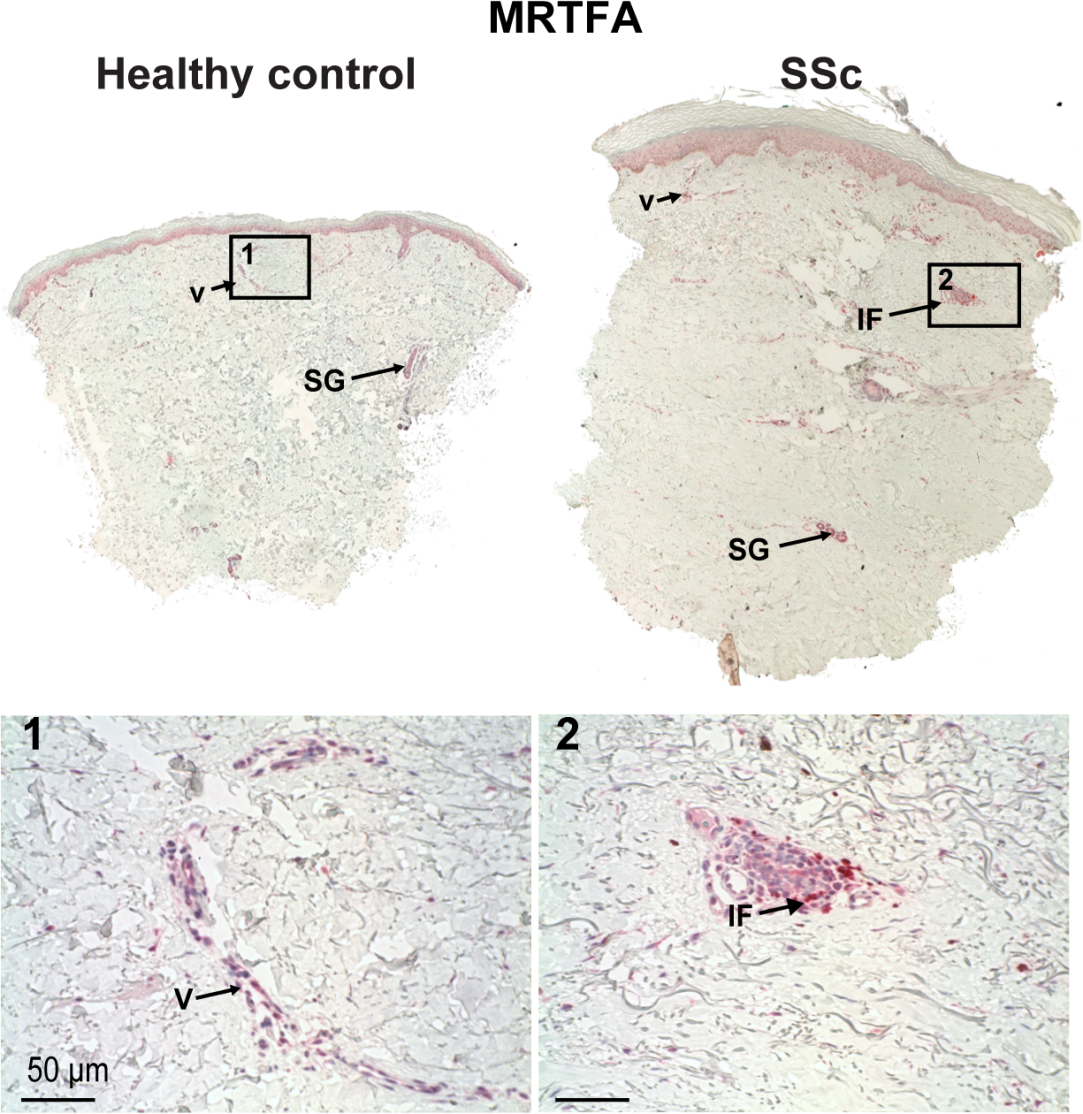
MRTF-A expression is more prominent in SSc skin then healthy control. MRTF-A staining (1:1000) of healthy control (left) and SSc (right) sections. Several pictures across the wound were merged to produce panoramas of whole sections. Original magnification 10X. Expression of MRTF-A is increased in SSc sections, with more seen in dermal cells, keratinocytes, and vasculature especially within inflammatory foci around small vessels in SSc. Arrow indicate sweat and sebaceous glands (SG), vascularture (V) or inflamatory foci (IF) stained with MRTFs. **1.** MRTF-A vascular cell staining in healthy controls or **2**. pervascular inflammtory foci in SSc. (30X).

**Fig 2:**
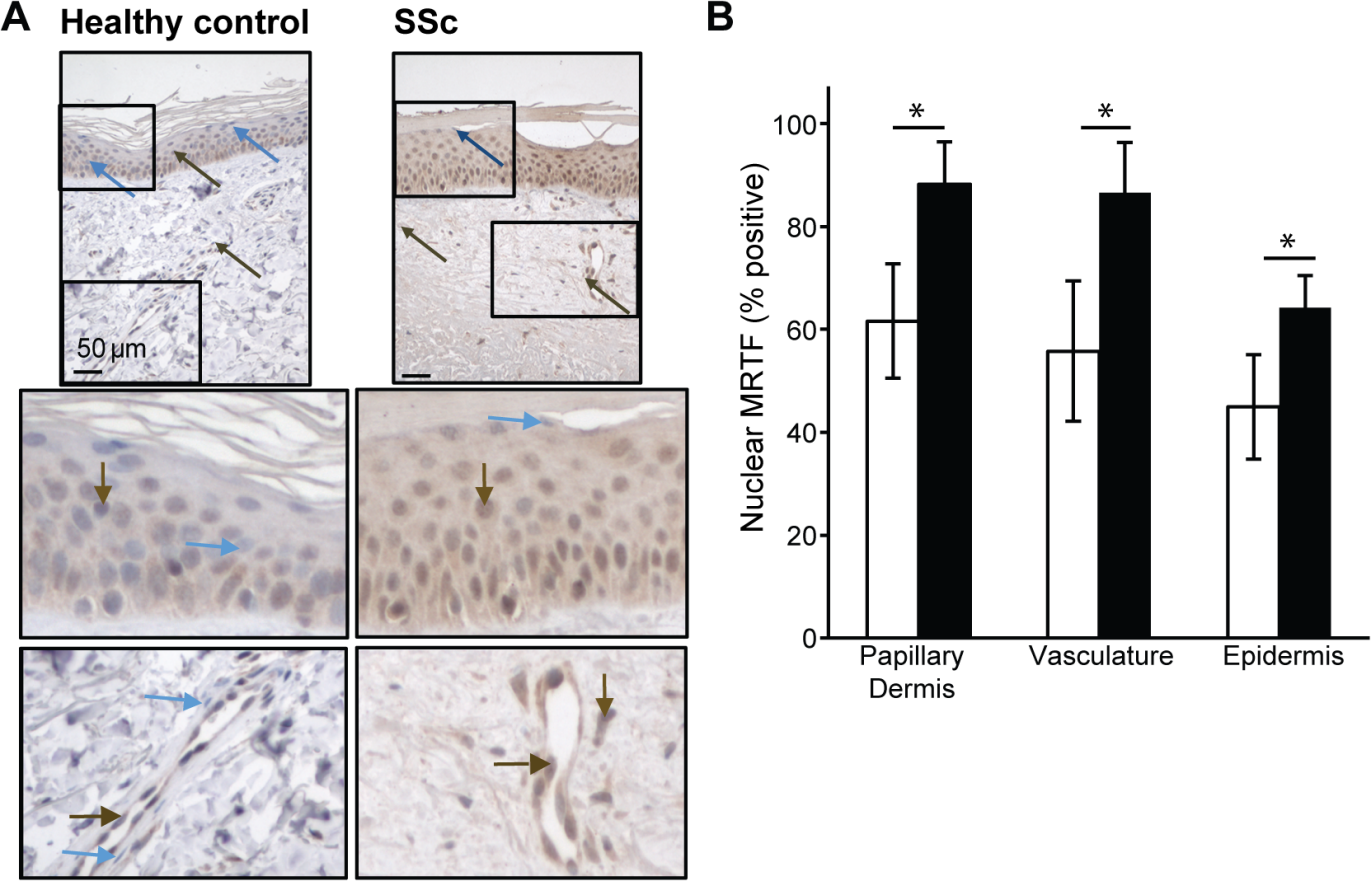
Nuclear MRTF-A expression is more prominent in SSc skin then healthy control. **A.** Representative pictures of healthy control and SSc sections of biopsy. Histological samples of human skin were stained with MRTF-A antibody (1:2000). Higher magnification of epithelium and small vessels (20X) in papillary dermis. Brown arrows = MRTF-A nuclei, Blue arrow = hematoxylin stained nuclei without MRTF-A. Graphical representations of % nuclei in epidermis, vasculature, and interstitial cells in papillary dermis. Histological samples of 5 healthy control and 9 scleroderma human skin were evaluated for nuclear staining. B. Total cells with MRTF-A nuclear localization in the epidermis, vasculature, and interstitial papillary dermal layers were counted and compared with the total amount of nuclei in the epidermal/papillary dermal layer. White bars = healthy controls, Black bars = SSc (* = p<0.01 using nonparametric Mann-Whitney U, two-tailed).

**Fig 3:**
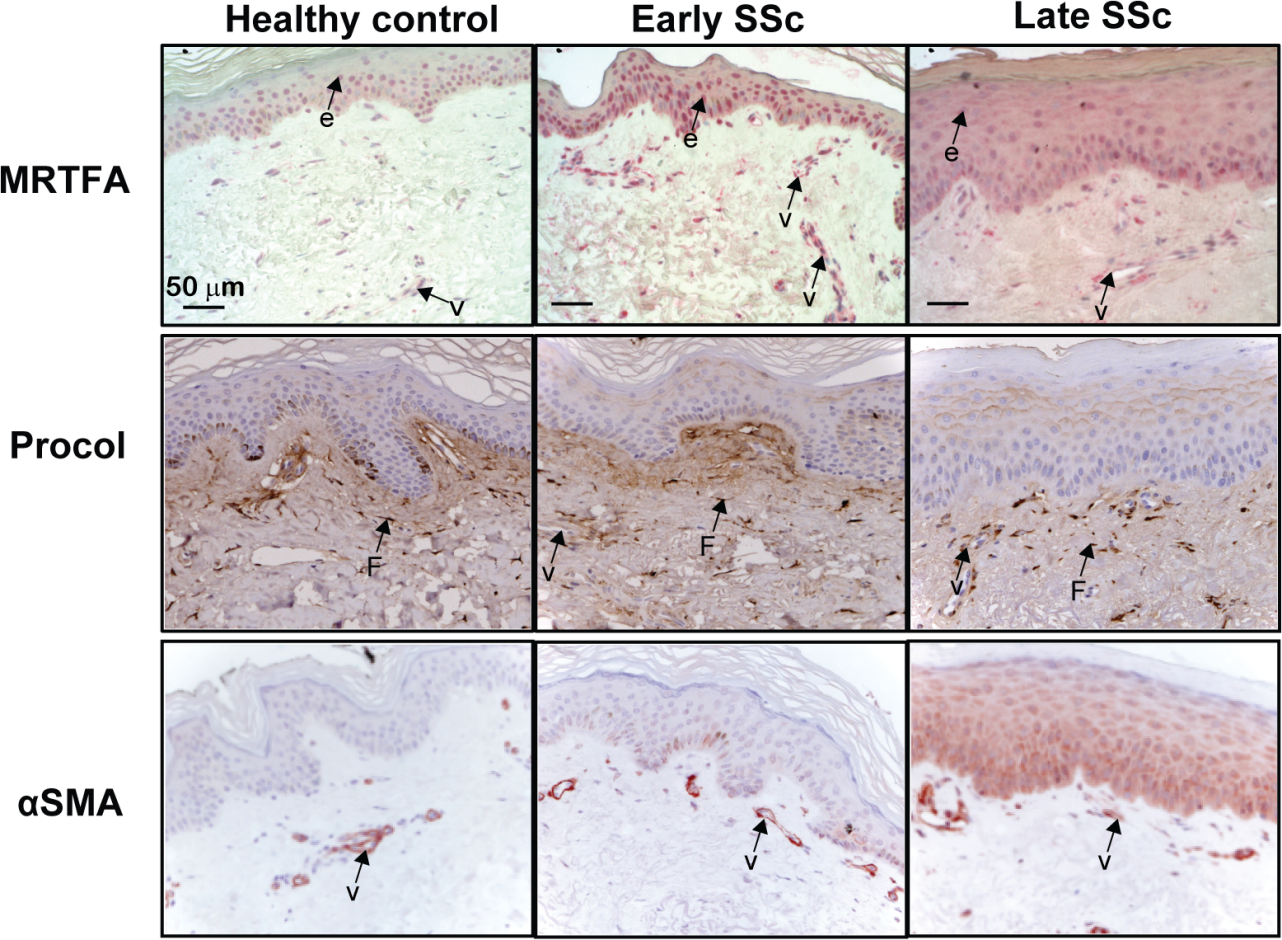
Increased expression of MRTF-A in the SSc epidermis and at the epidermal dermal junction in established SSc correlates with increased intracellular procollagen, and SMA. The expression of MRTF-A, procollagen type I, and SMA was detected by immunohistochemical staining in the epidermis and papillary dermis of early and established SSc patients and controls healthy control patients. Arrows point to staining in vascular cells (V), epidermis (e), and fibroblast (F).

**Fig 4:**
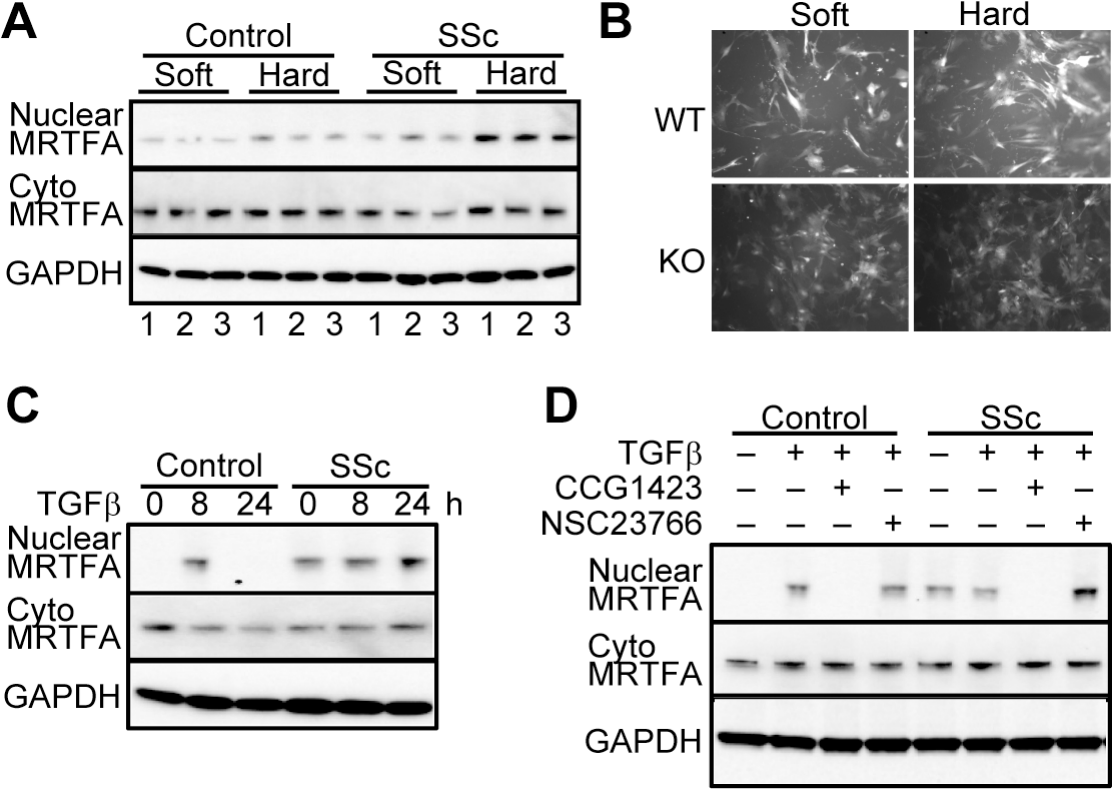
Stiff surfaces and TGFβ induce MRTF-A nuclear accumulation. SSc and control dermal fibroblast lines (both N = 3) were cultured on 6 well plates with collagen type I coated soft substrates (5 kPa Softwell), or hard substrate (50 kPa). **B. Induction of collagen transcription on hard surfaces requires MRTF-A.** Mouse fibroblasts from wild type (WT) and loss-of-function (KO) mice with collagen promoter driving GFP were cultured on fibronectin coated soft and hard surfaces. Pictures taken 24 hours after plating. Fluorescence was quantified using ImageJ. The numbers of cells in each image was counted (S4 Fig) to determine the luminance per cell. **C.&D.** Normal control fibroblasts and SSc fibroblasts were cultured without serum for 16 hours then treated with TGFβ (4 ng/ml) for 0, 8 and 24 hours or treated with either saline or TGFβ (4 ng/ml) with or without CCG1423 (10μM) or NSC2376 (50 μM). Proteins (20 μg) from cytoplasm and nuclei were extracted using NE-PER Nuclear Protein Extraction Kit and separated on 4–12% gradient gel and visualized using MRTF-A antibody.

**Fig 5:**
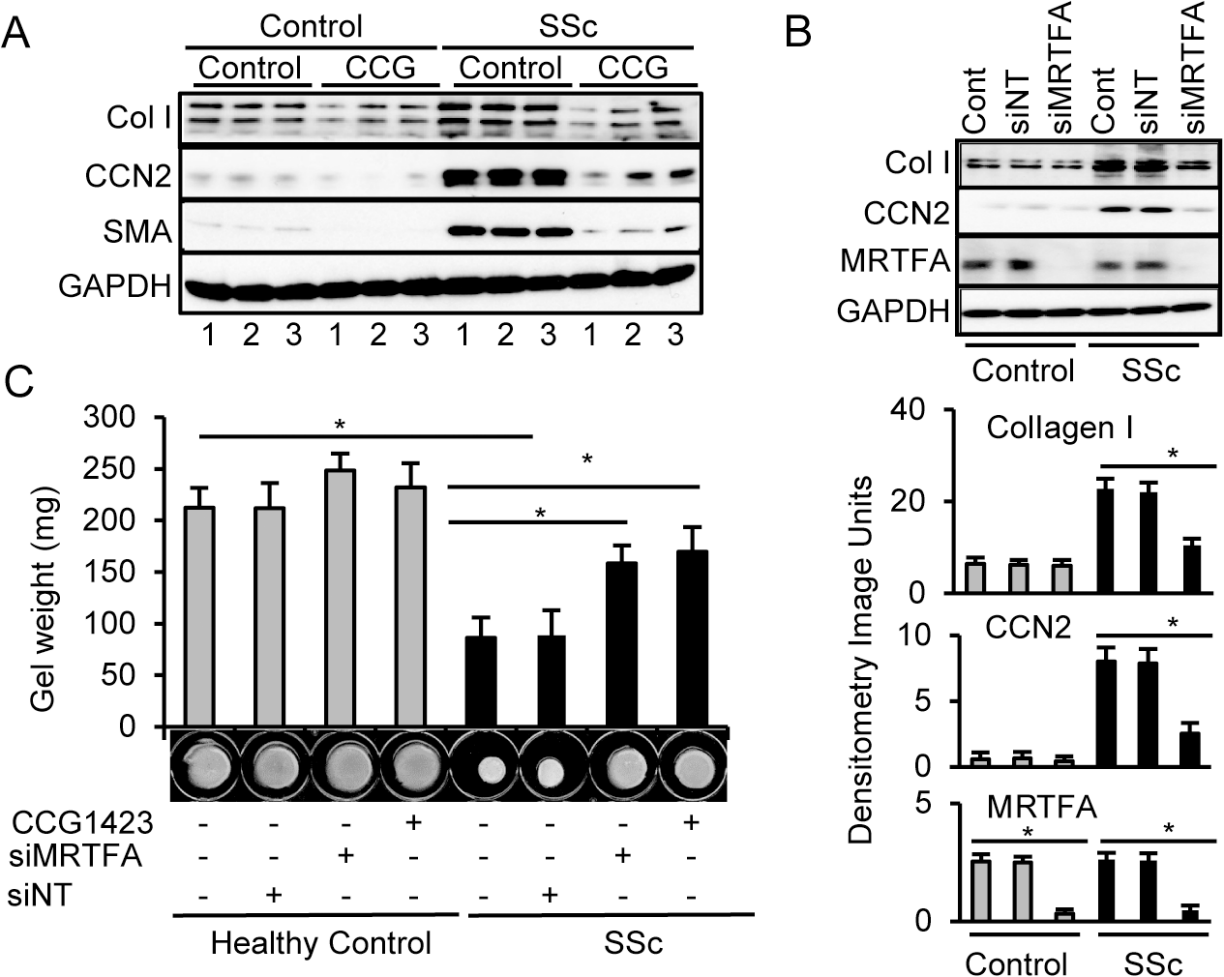
Enhanced type I collagen and CCN2 expression as well as collagen gel contraction in scleroderma fibroblasts is dependent on MRTF-A pathway. **A. The MRTF-A inhibitor, CCG-1423, blocks collagen and CCN2 synthesis.** Healthy control fibroblasts (Control) and scleroderma fibroblasts (SSc) from 3 independent isolates were cultured with or without the MRTF-A inhibitor CCG-1423 (10 μM). Basal CCN2 and type I collagen was increased in scleroderma cells and inhibited by CCG-1423. **B. Knockdown of MRTF-A by siRNA blocks collagen and CCN2 synthesis in SSc fibroblasts.** Control and SSc fibroblasts were treated with MRTF-A siRNA, (siMRTFA), a non-target siRNA (siNT), or vehicle (Cont). **C. Loss or inhibition of MRTF-A blocks contraction of collagen floating gels.** Knockdown (siMRTFA) and inhibition (CCG-1423) of MRTF-A partially blocks collagen floating gel contraction by SSc fibroblasts. * = p<0.05.

Boston University Medical Center and the UCL, Royal Free Hospital Institutional Review Board (IRB) approved all human studies performed at each location. All scleroderma patients fulfilled the American College of Rheumatology criteria for disease and were classified according to internationally accepted criteria [46]. All study subjects and healthy volunteers gave written informed consent. Punch biopsy samples (3 mm Boston, 4 mm London) of the skin over the dorsal midforearm were collected and prepared for histology at Boston University by DermPath Core, part of the National Scleroderma Center (Figs 1 and 2, S1 and S2 Figs). For the London Studies, conducted under the Royal Free Hospital IRB, patients were classified as early SSc if within the first 2 years of disease onset, defined by the appearance of the first non-Raynaud’s manifestation. Patients with disease duration above 2 years were classified as established as previously described [47]. Punch biopsy samples were prepared for histology by pathology at UCL Medical School (Fig 3) or placed in media for cell culture (Figs 4 and 5). Patients were not receiving immunosuppressive medication or corticosteroids at the time of biopsy if cells were cultured.

**Fig 6:**
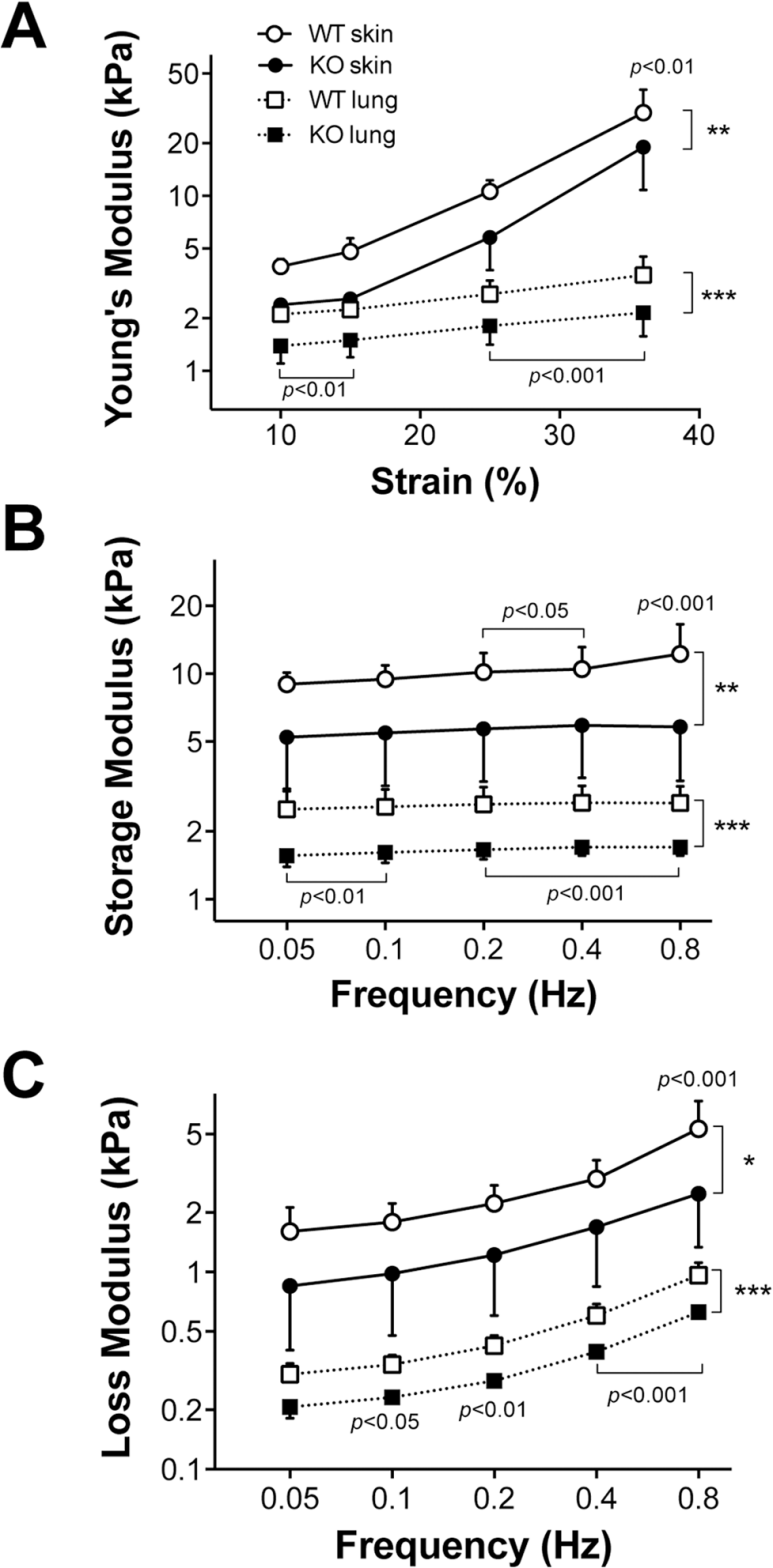
Mouse KO skin and lung are less stiff than WT skin and lung. A 3x1x1 mm strip of dorsal skin or lung tissue was placed longitudinally into a computer-controlled dual-mode lever arm force transducer system and stretched intermittently. **A.** Young’s module plotted at each strain. The Young’s module is the slope of the stress-strain curve. **B.** Dynamic stretch storage modulus describes the ability of the material to store elastic energy during the loading phase of a cyclic stretch **C**. Dynamic stretch loss modulus—The amount of energy lost (usually as heat) during a cycle. Vertical brackets denote the overall group differences using 2-way repeated measure ANOVA (*: *p*<0.05, **: *p*<0.01 and ***: *p*<0.001) whereas horizontal brackets show Tukey’s post hoc differences between WT and KO at the same strain (Panel A) or frequency (Panels B and C). For the Young’s and loss moduli of lung tissue, there are also significant interactions between strain and group (panel A) as well as frequency and group (panel C, p<0.05). For the skin, there is a significant interaction between frequency and group only for the loss modulus (p<0.05).

**Fig 7:**
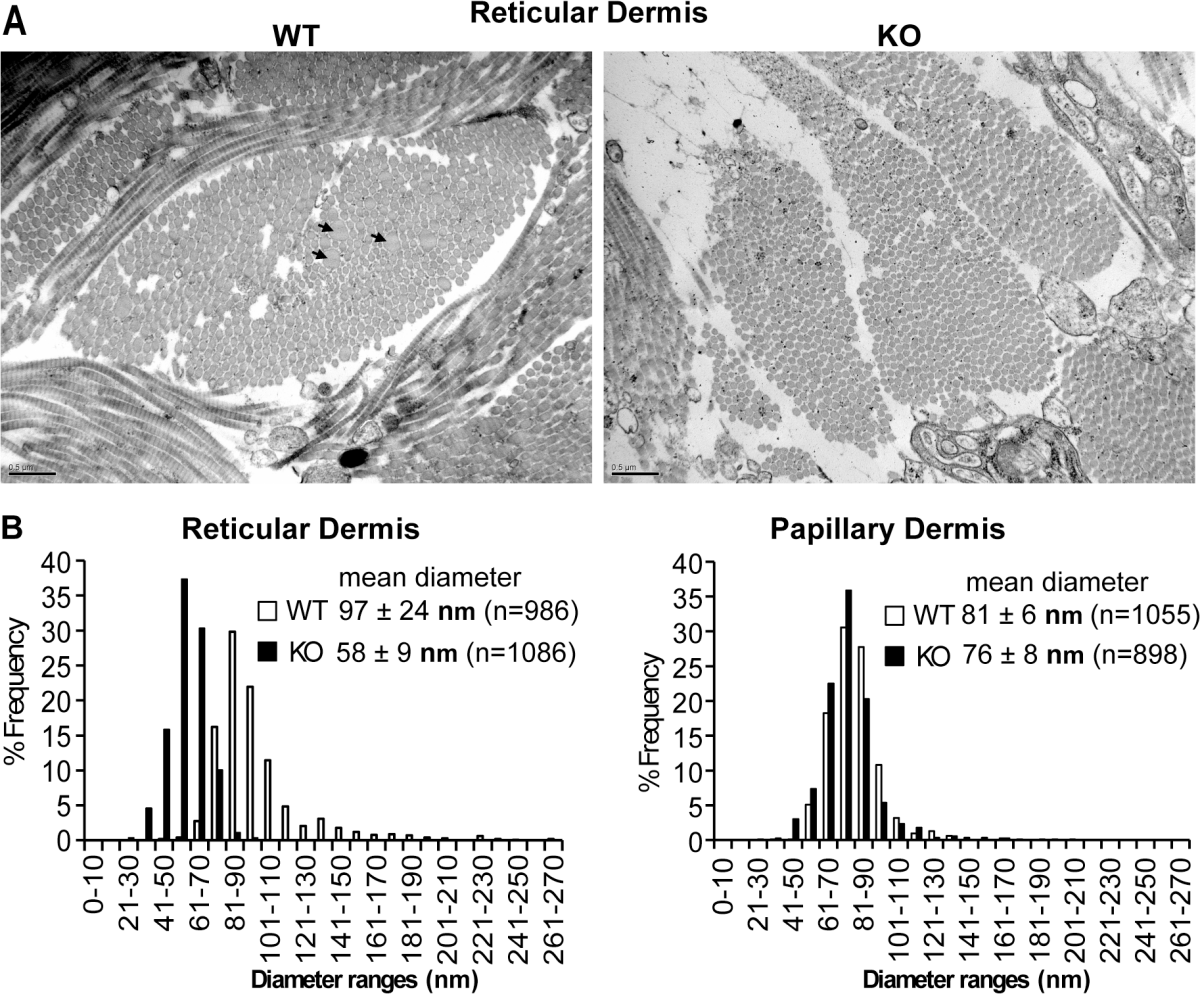
Fibril diameters in the KO reticular dermis are smaller and more uniform than WT fibrils. **A.** Representative micrographs of skin in deep dermal areas near subcutaneous fat. (Scale bar = 0.5 μm) **B.** Histogram of the frequency of collagen fibrils with a given diameter range from WT (white bars) and KO (black bars). At least 200 fibrils from 3 WT and 3 KO animals at 5 months of age were used for analysis. P<0.05.

The Boston University School of Medicine Institutional Animal Care and Use Committee (IACUC) approved all animal husbandry and experiments for isolation of cells and tissue (Figs 4B, 6 and 7). The UCL, Royal Free Hospital Comparative Biology Unit (CBU) Ethics and Welfare Committee approved all the London animal handling for cell culture of mouse dermal cells (S3 Fig).

### Histology

Biopsies of human skin were fixed in 4% paraformaldehyde embedded in paraffin, sectioned (5 μm) and stained using standard techniques. Sections were incubated with antibodies against MRTF-A (Santa Cruz C-19), MRTF-B (Sigma Prestige, USA), procollagen (Abcam, ab64409), and SMA (Sigma, USA) developed with alkaline phosphatase red reagent or horseradish peroxidase with DAB (3, 3’-diaminobenzidine) and counter stained with hematoxylin. For MRTF-A antibody, sections were treated for antigen retrieval at pH6, secondary antibody with alkaline phosphatase and counter stained with hematoxylin. MRTF-A localization to the cell nucleus was quantified by counting of immunostained (brown) nuclei versus hematoxylin stained nuclei (blue) per high power field. Nuclei were counted in 5 slides for each individual (N = 5 healthy controls, N = 9 SSc) and expressed as percentage of MRTF-A positive nuclei, mean and SEM for controls and SSc patients. Procollagen staining in cells was quantified by counting the number of brown stained cells in the papillary dermis of healthy controls (N = 4), early SSc (N = 3) and late SSc (N = 3). For transmission electron microscopy, samples were fixed using standard methods [48]. At least >200 fibrils from five different micrographs (40,000 magnification) within the dermis of each sample (N = 4 KO, N = 4 WT) were measured using ImageJ (NIH) to calculate an average fibril diameter.

### Cell culture

The MRTF-A loss of function mice (kindly supplied by Eric Olson) [44] were mated to mice transgenic with COL1A1 3.6 kb promoter driving GFPtopaz in C57/Bl6 background (kindly supplied by David Rowe)[49]. Lung cells from mice were extracted as described [19] and cultured at 60% density on fibronectin coated polyacrylamide gels in 6 well dishes [50] (Fig 4B). Five pictures were taken of each well and the numbers of cells and pixel density was quantified using ImageJ software. A second mouse colony was established at Royal Free Hospital London approved by the CBU Ethics and Welfare Committee according to European laws and regulations for the use and protection of vertebrate mammals for experimentation and other scientific purposes. Mouse and human dermal cell culture were isolated in London as described [51]. Fibroblasts were maintained in Dulbecco’s modified Eagle’s medium (DMEM; Gibco, Grand Island, NY) supplemented with 10% fetal bovine serum (Atlanta Biologicals or Gibco), 1% penicillin/streptomycin and incubated in 5% CO_2_ at 37°C. For some experiments cells were treated with TGFβ (R&D Systems) or grown on collagen type I coated polyacrylamide plates at 5 and 50 kPa (Softwell Collagen, Matrigen Products)(Figs 4A, 4C, 4D and 5).

### siRNA knockdown

Specific siRNA recognizing MRTF-A was purchased as predesigned siRNAs to MRTF-A (Dharmafect SMARTpool On TARGETplus MKL1 siRNA) alone or with a recommended non-target control siRNA (Ambion, Warrington, UK). Normal and SSc fibroblasts were transfected using Silencer siRNA Transfection II Kit (Ambion, Applied Biosystems, Warrington UK) with 60 nM siRNA. Cells were then used in floating collagen gel assay and for protein determinations by Western analysis.

### Floating collagen gel contraction assay

Experiments were performed as described previously [52]. Briefly, collagen/cell suspension (80,000 cells with 1.2 mg/ml collagen (Vitrogen)) was added to each bovine serum albumin coated wells (24-well plates). After polymerization, gels were detached from wells. In some experiments, CCG-1423 was added to medium. Contraction of the gel was quantified by loss of gel weight and decrease in gel diameter over a 24-h time period.

### Western analysis

Fibroblasts from monolayer culture were collected and lysed with 8 M urea and 1% SDS sample buffer or nuclei and cytoplasm was separated using NE-PER (Thermo Scientific). Proteins were quantified (Bradford, Bio-Rad, Hercules, California, USA), and equal amounts (25 μg) were subjected to SDS/PAGE using 4% to 12% polyacrylamide gels (Invitrogen, Paisley, UK). Gels were blotted onto nitrocellulose, and proteins were detected using anti-CCN2 (Abcam, UK), anti-SMA (Sigma, USA), anti-procollagen (Millipore, UK) and an enhanced chemiluminescence (Amersham, Little Chalfont, UK). Densitometry was performed using Quantity One software (Bio-Rad, Hercules, California, USA).

### Measurement of mechanical properties of skin and lungs

Dorsal skin from WT (N = 6) and aged-matched MRTF-A KO mice (N = 6) were shaved and cut into strips (3x1x1 mm) and orientated parallel to the spine. Lung tissue strips were obtained from the same animals. Uniaxial quasi-static stress-strain curves and oscillatory stress-strain loops were obtained as previously [53]. The strips were preconditioned by applying three triangular waves stretching the samples up to 40% strain at a rate of 0.75%/s. After a 5-min equilibration period, a quasi-static stress-strain curve was taken followed by dynamic measurements between 0.05 and 0.8 Hz with 40% strain for seven cycles. Force and length data were recorded and converted to stress and strain, respectively, using the dimensions of the strips.

### Statistics

The statistical significance was assessed by nonparametric Mann-Whitney test, ANOVA with Scheff post-hoc or Tukey post-hoc or paired t-test. P≤ 0.05 was considered as statistically significant.

## Results and Discussion

### MRTFs are up-regulated in multiple cell lineages within SSc skin

Since MRTF-A translocates to nucleus in response to mechanical stiffness, which is increased in SSc skin, the expression and location of MRTF proteins were examined in patients with diffuse SSc. MRTF-A was more widely expressed in SSc skin then in healthy controls as judged by immunochemistry (Figs 1, 2, and 3). Antigen peptide blocking and IgG controls demonstrated that MRTF-A staining was specific (S1 Fig). MRTF-A staining was most obvious in cytoplasm of epidermal derived cells such as keratinocytes, sebaceous glands and sweat glands in normal tissue biopsy which was increased in most SSc patients (Fig 1).

SSc disease starts with an initial vascular damage and subsequent inflammatory cell infiltrations surrounding small blood vessels in the papillary dermis [54]. Normal skin had MRTF-A staining in small vessels (Fig 1A, insets) suggestive of endothelial or pericyte expression within the normal microvasculature in the papillary dermis (Figs 1A and 2). MRTF-A in endothelial cells activates the adhesion molecule intercellular adhesion molecule 1, (ICAM-1), and endothelin through the inflammatory molecule nuclear factor kappa B (NFκB) subunit p65 and epigenetic modulating enzymes [55, 56]. Both these MRTF-A targets are increased [57] and function in SSc [58, 59]. MRTF-A also mediates TGFβ-induced endothelial to mesenchymal transition [30] which is important in SSc [60]. In SSc dermis with inflammatory foci, there is extensive MRTF-A staining (Fig 1A insets). Inflammatory foci contains activated macrophages [61]. In cultured macrophages, MRTF-A mediates synthesis of pro-inflamatory molecules such as tumor necrosis factor (TNFα) [62, 63] that are increased in SSc early disease [64]. MRTF-A levels increase in the vasculature following atherosclerotic injury [21] and controls vessel growth and maturation through CCN1 and CCN2 [65].

### MRTF-A is predominantly nuclear in SSc skin

Since MRTF-A translocates to the nucleus to act as a transcription co-activator, the percentage of nuclear MRTF-A was calculated for multiple cell types (Fig 2). In healthy control skin (N = 5), keratinocytes (45%±6), fibroblasts (61%±11) and vascular (56%±14) cells in microvessels had nuclear MRTF-A staining in the papillary dermis (Fig 2). There was significantly more nuclear MRTF-A staining in SSc keratinocytes (N = 9) (64%±10), dermal fibroblasts (88%±8), and vascular cells (87%±10) (Fig 2). Compared with healthy controls, the SSc dermis contained extensive MRTF-A staining within inflammatory foci in the perivascular area surrounding vessels (Fig 1A).

### Keratinocyte activation in SSc is correlated to increased nuclear localization of MRTF-A

Previous data from our group demonstrated that SSc epidermis is activated with delayed keratinocyte differentiation and changes resembling those seen during wound healing [47, 66, 67]. Here we show MRTF-A nuclear staining of healthy control epidermis was concentrated in the basal area in normal keratinocytes (Fig 3, top panels). The keratinocytes in SSc exhibited increased nuclear expression of MRTF-A extending into the upper regions of the epidermis suggesting activation of keratinocytes. A key role of MRTF-A in keratinocyte biology was shown previously by Watt and colleaques, who demonstrated that basal keratinocytes proliferate until encountering a physically constrained environment whereupon they release MRTF-A from actin and signal to induce involucrin and the terminal differentiation program [68]. Our recent data suggests that classical terminal differentiation markers are extended downwards to the spinous layer keratinocytes, with all but basal cells positive for involucrin [47]. Characteristically, SSc cells are taking on an altered pathway of differentiation, expressing activation markers, wound related cytokeratins, and premature expression of maturation markers such as involucrin, a known target of MRTF-A [47]. In addition, MRTF-A is an important downstream signal during TGFβ induced-EMT [28]. Since SMA is a direct target of MRTF-A in EMT, the same patient’s slides were examined using a SMA antibody (Fig 3, bottom panels). Several late stage patients with highest levels of nuclear MRTF-A have SMA staining in the keratinocytes suggesting partial EMT. Furthermore, in SSc, the activated epidermal cells are able to release factors capable of inducing local dermal fibroblasts to become, CCN2 producing cells [47, 66, 67]. SSc keratinocytes themselves are capable of expressing S100A9 which induces fibroblast proliferation and to enhances fibroblast CCN2 expression [47].

### SSc cells within the epidermal-dermal juncture have increased nuclear MRTF-A and collagen

Our previous data demonstrates that Collagen type I is a direct transcriptional target of MRTF-A [19]. Therefore, sections were examined using a procollagen I antibody directed at the N-terminal propeptide. The staining was predominantly cytoplasmic consistent with local cells producing collagen. In late stage SSc when stiffness is highest, there were significantly more cells (2 fold) staining with collagen (Fig 3, middle panels). The papillary dermis contains activated fibroblasts producing collagen with no SMA staining other than vascular cells (Fig 3, bottom panels, and S2 Fig). ECM collagen staining at the epidermal-dermal border was noted in healthy and early SSc (Fig 3, middle panels) presumably newly synthesized small collagen fibrils maintaining the N-terminal propeptide [69]. Small diameter type III/type I fibrils (20–40 nm) containing N-terminal propeptides are normally found at the epidermal/dermal juncture [69]. In late stage SSc, there was less N-terminal peptide staining in the ECM (Fig 3). This agrees with earlier studies that reported increased numbers of larger diameter (80–120 nm) collagen fibrils with no N-terminal peptides in SSc papillary and reticular dermis [10].

### Reticular dermis in SSc skin contain cells with nuclear MRTF-A that express SMA

In later stages of SSc, collagen accumulates leading to increased dermal thickening with a loss of microvasculature and fibrosis of the reticular dermis and subcutaneous adipose tissue. In order to examine the correlation of nuclear MRTF-A with SMA expression that identify myofibroblasts, a series of patient’s biopsies were stained for MRTF-A and consecutive slides were stained for SMA. Dermal SMA+ myofibroblasts in established SSc were primarily located in reticular dermis with strong SMA+ and MRTF-A nuclear staining (S2 Fig). MRTF-A regulates myofibroblast differentiation through mechanotransduction [33] which is a process whereby a living cell converts mechanical cues to biochemical signals maintained by a physical continuity between the ECM/integrin/cytoskeleton/nuclear matrix structures [70]. Fibroblasts in the papillary dermis exhibited nuclear MRTF-A staining, but limited SMA staining other than microvessels. Erector pili muscles were strongly SMA+ with a subset of nuclear MRTF-A staining muscle cells (50% in normal muscle, 90% in SSc). Epithelial structures (sebaceous and sweat glands) were stained in the cytoplasm with MRTF-A, with little staining of SMA. In the epidermal-dermal juncture region, MRTF-A staining was present in the endothelial cell nuclei in small papillary vessels next to the cells staining for SMA (pericytes and/or smooth muscle cells). In established disease, myofibroblasts are present primarily in the reticular dermis not in the papillary dermis.

### MRTF-A translocates to the nucleus on stiff fibrotic-like matrices or by TGFβ

Activation of MRTF-A may be part of a feed forward mechanism whereby cells sense stiff matrix and produce more ECM during progression of fibrosis in SSc [38]. Changes in mechanical environment were modeled *in vitro* by growing SSc and healthy control fibroblasts on soft or stiff collagen coated polyacrylamide gels (Softwell 5 kPa vs 50 kPa). The increased substrate stiffness enhanced nuclear translocation of MRTF-A in dermal fibroblasts (Fig 4A). The SSc cells had more nuclear MRTF-A on soft and stiff matrices than healthy control cells similar to the histology. Therefore, SSc fibroblasts express more nuclear MRTF-A even under conditions mimicking normal skin stiffness.

Most importanly, collagen expression and synthesis increases on stiffer matrices [71]. To examine whether MRTF-A controls collagen expression with stiffness, MRTF-A KO mice [44] were bred to transgenic mice with an integrated copy of 3.6kb Col1A1 promoter driving GFPtopaz [49] to measure fluorescence as previously described [19]. Isolated lung cells from WT and MRTF-A KO transgenic mice, produce less collagen which can be rescued by over-expressing MRTF-A [19]. In addition, the cells do not transcribe more collagen driven GFP in response to TGFβ [45]. Lung cells from WT and KO mice were grown on polyacrylamide gel substrates functionalized with fibronectin to encourage myofibroblast differentiation [50]. WT cell proliferation, collagen production and transcription were increased on surfaces with similar stiffness to fibrotic tissue. Without MRTF-A, collagen transcription and proliferation were not increased on fibrotic-like surfaces (Fig 4B and 4S). The intensity of fluorescence per cell decreased in KO cells on fibrotic-like matrices. These KO cells did not modulate their collagen transcription on stiff matrix (Fig 4B). Others have also demonstrated that MRTF-A directs myofibroblast differentiation and SMA expression on stiff matrices [27]. Our data suggests that the increase in collagen on stiffer matrix is MRTF-A dependent.

Since TGFβ enhances MRTF-A translocation in cell specific manner [28, 72], SSc and control fibroblasts were plated on soft matrix (5 kPa) and treated with TGFβ to determine if fibrotic and normal cells differ. Nuclear MRTF-A was present at very low levels in control fibroblasts, but was strongly present in scleroderma cells. TGFβ stimulated nuclear accumulation of MRTF-A levels after 8 hours in both SSc and healthy controls which reverted back to baseline levels by 24 hours (Fig 4C). Our results suggest that baseline amounts of nuclear MRTF-A are elevated in SSc cells and TGFβ-induced translocation of MRTF-A occurs in healthy control fibroblasts. Since the pharmacological inhibitor, CCG-1423, blocks MRTF-A nuclear translocation [20, 21], SSc and healthy control cells were treated with CCG-1423 to determine if it would inhibit TGF-β-induced translocation of MRTF-A. As shown in Fig 4D, CCG-1423 blocked TGF-β-induced MRTF-A translocation (Fig 4D). Rac1 is essential for repair mechanism [73] and knockout of Rac1 decreases bleomycin induced fibrosis [74]. Since Rac1 signaling has been implicated in MRTF-A signaling [75], cells were also treated with a Rac inhibitor, NSC23766 [76]. However, Rac1 inhibition did not alter MRTF-A nuclear translocation.

### CCG-1423 and MRTF-A knockdown decreases collagen, SMA, and CCN2 expression in SSc cells

Cultured early passage dermal fibroblasts from patients with SSc have a basal pro-fibrotic phenotype and maintain their ability to synthesize more collagen and CCN2 in tissue culture [77, 78]. In order to determine whether the phenotype is dependent on MRTF-A, control and SSc dermal fibroblasts were treated with CCG-1423 to determine if inhibition of MRTF-A nuclear translocation would decrease collagen, SMA and CCN2, an important profibrotic cytokine. Compared to controls, basal levels of type I collagen, SMA, and CCN2 were elevated in SSc fibroblasts. CCG-1423 decreased collagen, SMA, and CCN2 levels in both control and SSc fibroblasts (Fig 5A). Knocking down MRTF-A with siRNA also reduced collagen and CCN2 expression, especially in SSc fibroblasts, similar to the pharmacological inhibitor (Fig 5B). The enhanced synthesis of ECM molecules by SSc cells was dependent on nuclear MRTF-A expression and nuclear localization.

### MRTF-A is required for collagen gel contraction by SSc cells

An important function of myofibroblasts is contraction [78], which was measured by growing cells in floating 3D collagen gels. As shown previously [52], SSc fibroblasts contracted the collagen gels significantly more than healthy control fibroblasts (Fig 5C) through stimulation of the TGFβ activation pathway [79, 80]. The MRTF-A siRNA and CCG-1423 blocked collagen gel contraction by SSc fibroblasts but did not alter contraction of healthy controls. Isolated fibroblasts from WT mice contracted the gels significantly more than KO fibroblasts (S3 Fig) as shown by others [81]. Since TGFβ increases contraction, gels were treated with and without TGFβ. TGFβ treatment increased contraction of normal cells, but not KO cells (S3 Fig). When MRTF-A was either inhibited by CCG-1423 or knocked down by MRTF-A siRNA in WT fibroblasts, TGFβ-induced contraction was diminished, whereas TGFβ-induced contraction was not significantly altered in MRTF-A KO fibroblasts. This strongly suggests that nuclear MRTF-A is required for fibrotic myofibroblast function and the inhibitor not only alters collagen and CCN2 synthesis but also collagen gel contraction.

### Loss of functional MRTF-A decreases mechanical stress and stiffness of skin and lungs

Since collagen synthesis by KO cells is lower than WT cells [19] and MRTF-A translocation is induced by mechanical stiffness, the biomechanical properties of dermal and lung tissue in KO mice were tested. Skin and lung tissue strips obtained from MRTF-A KO and WT mice were quasi-statically and dynamically stretched. The KO mice developed less stress and showed lower stiffness than normal mice in both dermal and lung tissue during quasi-static stretch (Fig 6A). The Young’s modulus from KO dermis was 45% lower than WT at 36% strain (p<0.01). Dynamic oscillatory data confirmed that tissues from KO mice were significantly less stiff than tissues from WT mice with both storage (Fig 6B) and loss (Fig 6C) moduli increasing with frequency. These data suggest that MRTF-A not only responds to stiffness, but actually creates a stiffer ECM in multiple organs. Mice deficient in MRTF-A have a basal level of mechanical stress that is reduced in the dermis consistent with resetting of the mechanical stress-adhesion-contraction mechanism (Fig 6). Areas of increased stiffness correlates with areas rich in collagen [71].

### Dermal collagen fibrils in KO mouse are smaller and more uniform

Since the biomechanical data suggest that there is less collagen or looser arrangement of fibrils in the KO mice, skin was prepared for electron microscopy to examine collagen fibrils. The diameters of collagen fibrils from WT and KO were quantified from EM micrographs (Fig 7). Although the KO papillary dermis fibrils were not significantly different than the WT, collagen fibrils in the reticular dermis, deep in the dermis near the adipose tissue, were significantly smaller (KO mean 57 nm, WT 97 nm) and more uniform in size than wild type mice. The results of this study are remarkably similar to the matricellular protein secreted protein acidic and rich in cysteine (SPARC/osteonectin) null mouse where the deficiency of SPARC reduced the collagen fibril diameter with more uniform distribution in the reticular dermis and decreased the skin tensile strength [82]. In SSc with activated MRTF-A, collagen fibrils are 2–3 times more abundant with larger variations in fibril diameters [10]. The difference between reticular and papillary dermis may be an important distinction since reticular and papillary dermis cells may have distinct lineages [83]. In SSc dermis in established disease, the myofibroblasts are present primarily in the reticular dermis. The papillary dermis has cells with nuclear MRTF-A but without SMA other than vascular cells.

## Conclusion

Skin and organ based fibrosis in SSc remains a challenging condition to treat because once established, fibrosis is mantained by a feedforward loop of increased adhesion, contractility, and increased mechanical stress leading to further myofibroblast differentiation and matrix modification. MRTF-A is recognized as a major mechanosensitive signaling molecule that, following mechanical force, is released from cytoplasmic G-actin and translocates to the nucleus to activate gene transcription of multiple genes in a cell specific manner [15, 17]. Outside of the context of SSc, a number of published studies support the notion that MRTF-A signaling has an essential role in fibrosis in different disease settings including LPA induced peritoneal fibrosis and pulmonary fibrosis; as well as bowel; hepatic and renal fibrosis [84–89]. Our data, represents the first examination of MRTF-A expression in human skin and demonstrates that MRTF-A expression in the nucleus is increased in several cell types in vasculature, inflammatory foci and fibroblasts to activate multiple targets within the SSc skin. In Scc, through environment stress genetically predisposed individuals develop endothelial cell damage and activation, as well as perivascular infiltration of inflammatory immune cells into the affected dermis. Epidermal cells also become activated taking on a tissue repair phenotype. These cell populations induce resident fibroblasts via cross talk, leading to myofibroblast differentiation, contractility, and initiation of ECM modifications, which increase mechanical stress. MRTF-A links mechanical stress to a harmful feed-forward mechanism via the induction of pro-fibrotic/tissue repair genes. CCG-1423, an inhibitor of nuclear translocation of MRTF-A [20, 90], reduces contractility and suppresses fibrotic targets in SSc fibroblasts. It is possible that drug therapies which target MRTF-A signaling could uncouple the persistent fibrosis from mechanical stress in SSc tissues leading to resolution or improvement, or would improve the responses to immunosuppressive or targeted anti-inflammatory treatments.

## Supporting Information

S1 Fig SSc section stained with MRTF-A antibody compared to isotype IgG and blocking antibody controls indicate that antibody is specific. Histological samples of human skin of SSc patient was stained with MRTF-A antibody (Santa Cruz C-19) (1:2000), IgG control, or with antibody and blocking peptide. Sections were counterstained with hematoxylin. Brown arrow points to MRTF-A nuclei, Blue arrow points to nuclei without MRTF-A.
(TIF)

S2 Fig Serial sections were stained with MRTF-A and SMA. **A** Several pictures across the skin were merged to produce panoramas of whole sections. Original magnifications 10X. Arrows point to vascular (V), erector pilus muscle (M), fibroblast (F) and myofibroblasts (MF). Line on SSc sections represent the separation between papillary and reticular dermis. Boxes with numbers represent the higher powered pictures in B—D. **B. Higher magnification** (20X) of papillary dermis with small vessels. SMA staining in myofibroblasts below the dotted line. MRTF-A nuclear staining without SMA above the line other than is vasculature. **C**. Papillary/Reticular dermis with erector pilus muscle **D.** Reticular dermis of same patient with myofibroblasts and adipose tissue staining in SSc, but not in normal skin sections.
(TIF)

S3 Fig MRTF-A is required for collagen gel contraction in mouse cells. **A.** Collagen gel contraction was significantly reduced in MRTF-A deficient cultures. KO cells did not contract the collagen gel even with the addition of TGFβ. TGFβ increased collagen contraction in WT. CCG-1423 blocked the contraction in the WT but not in the KO. * = p<0.01 WT vs TGFβ treated WT, ** = p<0.005 KO vs WT control. **B. Knockdown of MRTF-A by siRNA blocks TGFβ-induced contraction in WT but not in KO cells**. siNT = non-target siRNA siMRTFA = MRTF-A siRNA.
(TIF)

S4 Fig Bright field pictures of fluorescent cells in Fig 4B. (TIF)
