# Supplementary figures and images for: A Role of Myocardin Related Transcription Factor-A (MRTF-A) in Scleroderma Related Fibrosis
Source: PLoS One. 2015 May 8;10(5):e0126015. doi: 10.1371/journal.pone.0126015 (PMC4425676; doi:10.1371/journal.pone.0126015)

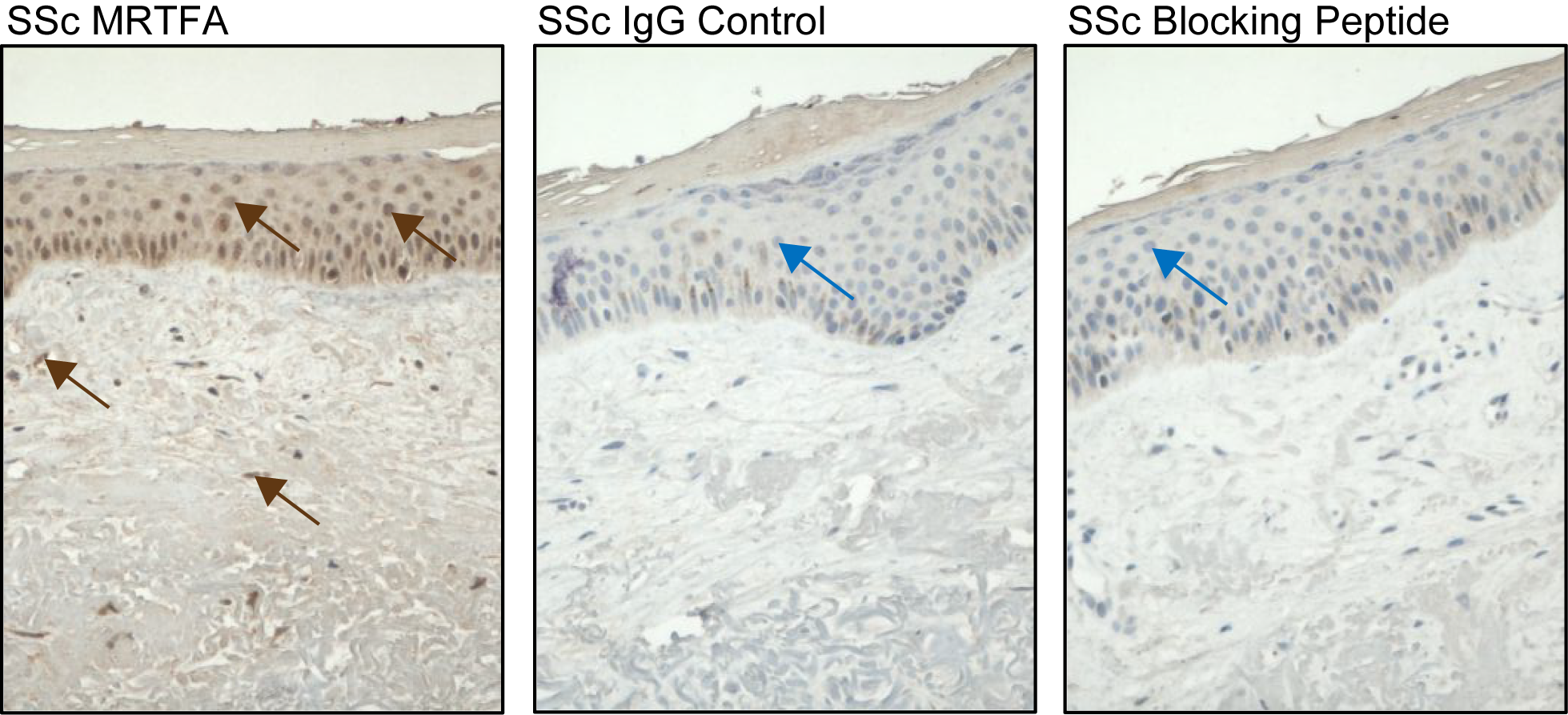

Supplement: S1 Fig — Histological samples of human skin of SSc patient was stained with MRTF-A antibody (Santa Cruz C-19) (1:2000), IgG control, or with antibody and blocking peptide. Sections were counterstained with hematoxylin. Brown arrow points to MRTF-A nuclei, Blue arrow points to nuclei without MRTF-A. (TIF) [file pone.0126015.s001.tif]

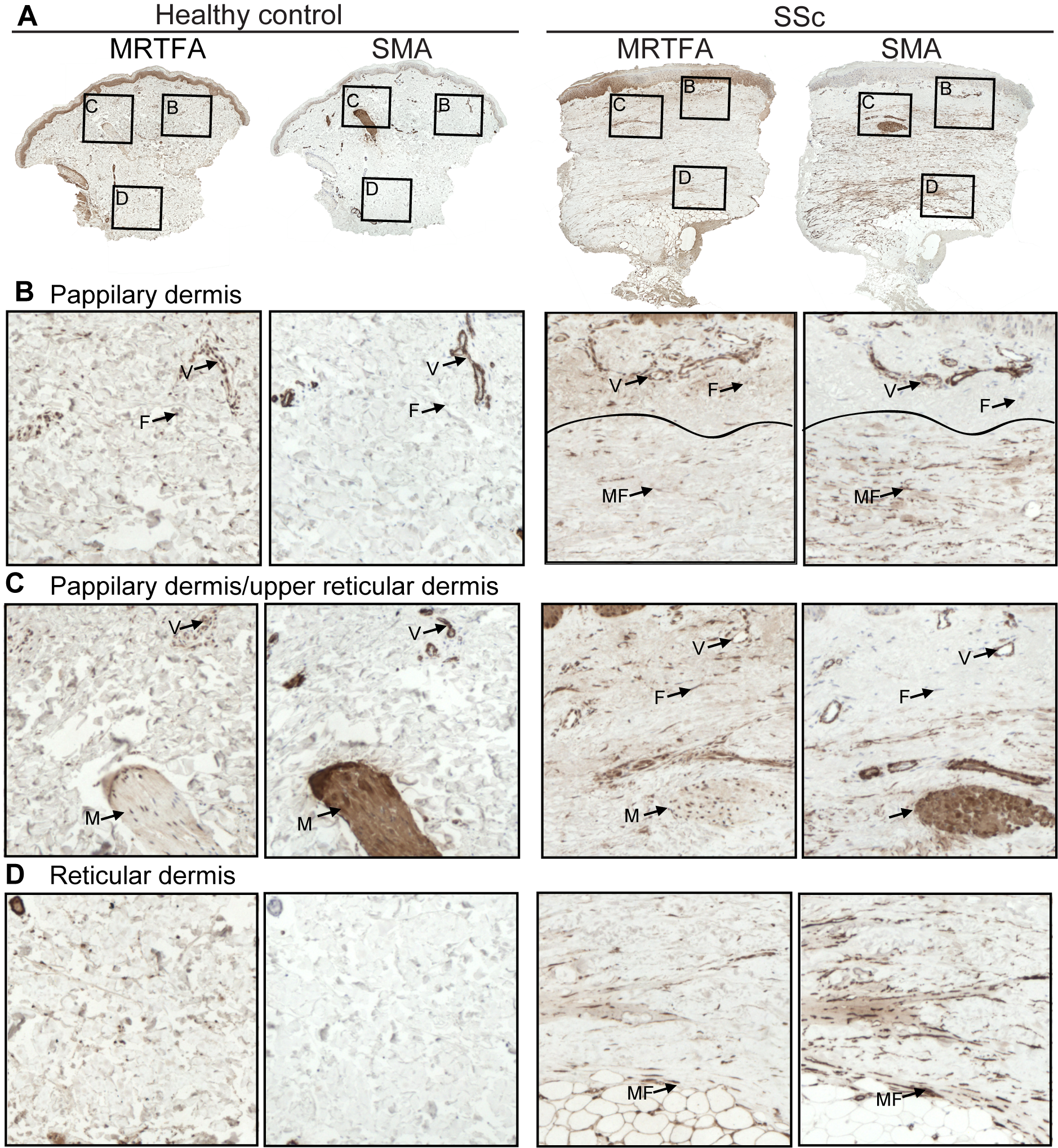

Supplement: S2 Fig — A Several pictures across the skin were merged to produce panoramas of whole sections. Original magnifications 10X. Arrows point to vascular (V), erector pilus muscle (M), fibroblast (F) and myofibroblasts (MF). Line on SSc sections represent the separation between papillary and reticular dermis. Boxes with numbers represent the higher powered pictures in B—D. B. Higher magnification (20X) of papillary dermis with small vessels. SMA staining in myofibroblasts below the dotted line. MRTF-A nuclear staining without SMA above the line other than is vasculature. C. Papillary/Reticular dermis with erector pilus muscle D. Reticular dermis of same patient with myofibroblasts and adipose tissue staining in SSc, but not in normal skin sections. (TIF) [file pone.0126015.s002.tif]

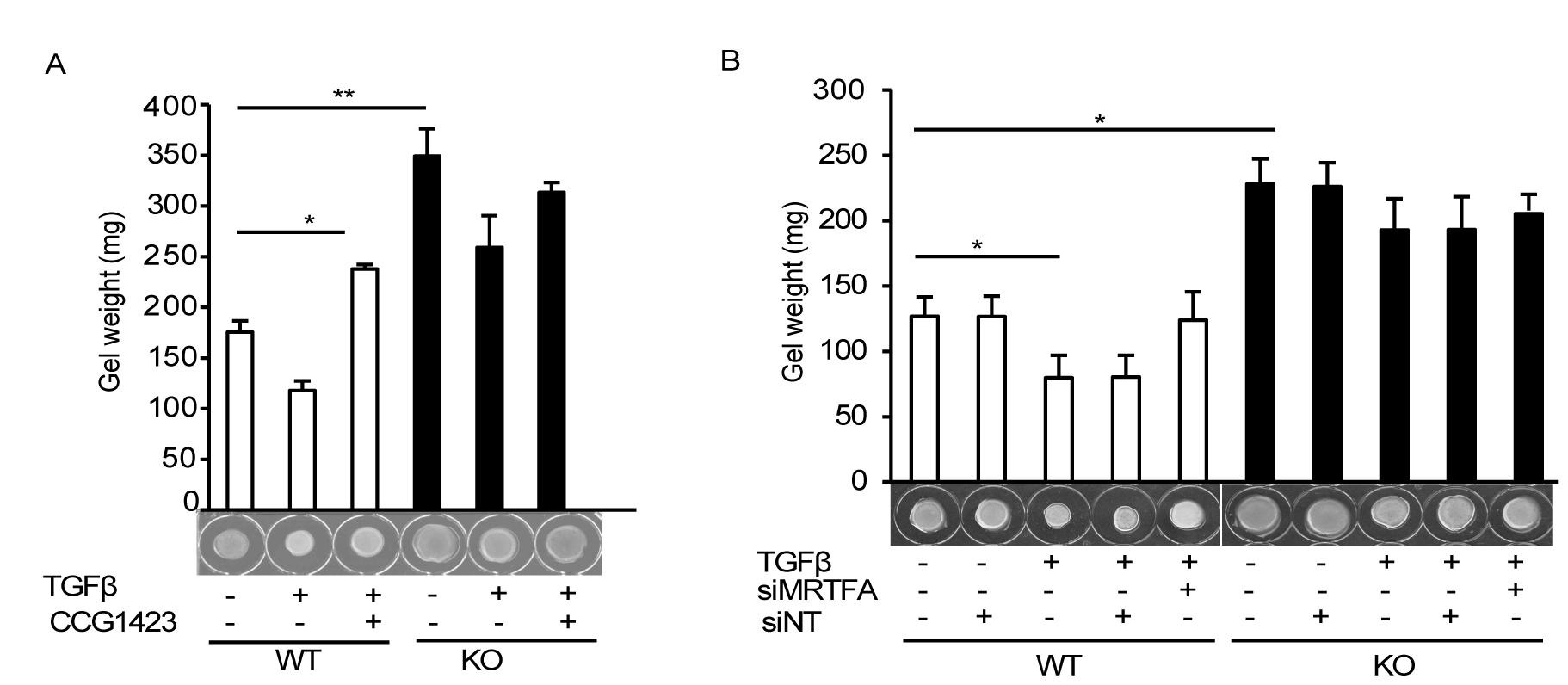

Supplement: S3 Fig — A. Collagen gel contraction was significantly reduced in MRTF-A deficient cultures. KO cells did not contract the collagen gel even with the addition of TGFβ. TGFβ increased collagen contraction in WT. CCG-1423 blocked the contraction in the WT but not in the KO. * = p<0.01 WT vs TGFβ treated WT, ** = p<0.005 KO vs WT control. B. Knockdown of MRTF-A by siRNA blocks TGFβ-induced contraction in WT but not in KO cells. siNT = non-target siRNA siMRTFA = MRTF-A siRNA. (TIF) [file pone.0126015.s003.tif]

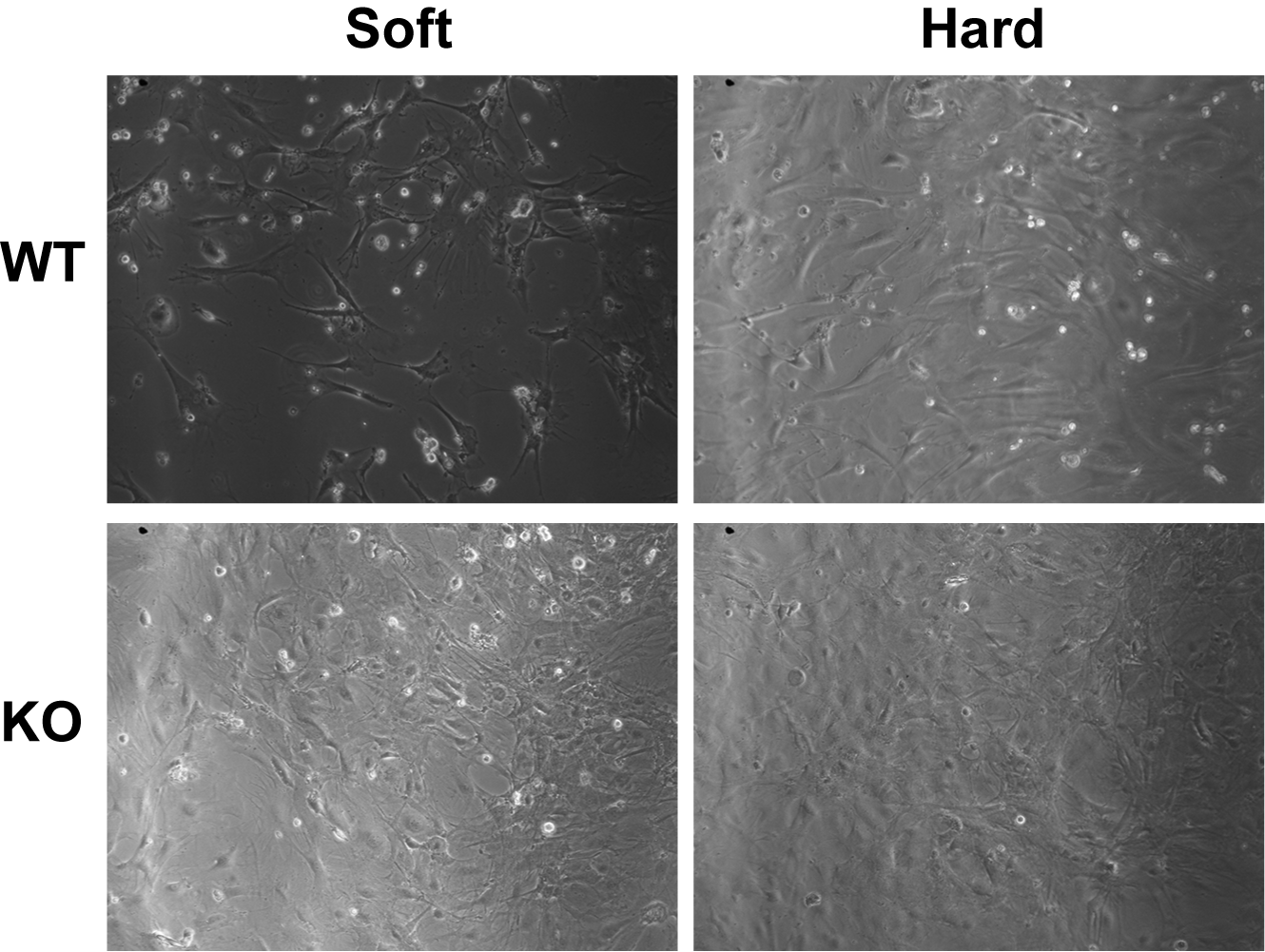

Supplement: S4 Fig — (TIF) [file pone.0126015.s004.tif]
